# Supplementary material for: Households’ expenditures for solid waste management services: Influencing factors and deep insight
Source: PLoS One. 2025 Jun 23;20(6):e0324085. doi: 10.1371/journal.pone.0324085 (PMC12184931; doi:10.1371/journal.pone.0324085)
Supplement: Attachment — Submitted filename: PONE-D-24-31637 (Referee).pdf [file pone.0324085.s002.pdf]

PLOS ONE

Households' expenditures for Solid Waste Management Services: influencing factors and deep insight

--Manuscript Draft--

**Manuscript Number:**	PONE-D-24-31637
**Article Type:**	Research Article
**Full Title:**	Households' expenditures for Solid Waste Management Services: influencing factors and deep insight
**Short Title:**	Determinants of waste disposal services
**Corresponding Author:**	Ghulam Mustafa, Ph.D<br>University of Education<br>Lahore, PAKISTAN
**Keywords:**	solid-waste; household; indigenous household survey; expenditures; Waste management
**Abstract:**	This study aims to document the existing solid waste collection and management system available to households in Pakistan and to explore the factors affecting households' cash payments for waste collection and disposal services. Robust least square regression is applied to household-level data from 16,155 households in the Pakistan Social Living Measurement Survey (PSLM) for 2019-2020. This method was chosen for its ability to handle outliers and provide more reliable estimates. On average, households pay PKR 214 (USD 1.01) per month for waste collection and disposal services. Households in Baluchistan and Khyber Pakhtunkhwa pay the highest amounts, while those in Sindh and Punjab pay less. Rural households pay more than urban households. Waste collection is primarily handled by private vans/carts, with door-to-door collection being the most common method. The municipality's role in waste collection at the doorstep is limited. Public bins are accessible to 83 percent of households, but their distant locations and infrequent emptying pose significant problems. These limitations highlight the need for improved municipal involvement and infrastructure. Results indicate that household income, doorstep waste collection via private van/cart, availability of bins or dumpsters, distance from waste disposal facilities, bin clearance duration, house ownership, dwelling type, and number of rooms significantly affect households' cash payments for waste collection services. To increase cash payments for waste collection services, waste management authorities should provide better and modern solid waste management systems. Upgrading existing systems can enhance households' willingness to pay for these services.
**Order of Authors:**	Ghulam Mustafa, Ph.D
	Naveed Hayat
	Bader Alhafi Alotaibi, PhD
	Abou Traore, PhD
**Additional Information:**	
**Question**	**Response**
**Financial Disclosure**<br><br>Enter a financial disclosure statement that describes the sources of funding for the work included in this submission. Review the submission guidelines for detailed requirements. View published research articles from *PLOS ONE* for specific examples.	Yes

Powered by Editorial Manager® and ProduXion Manager® from Aries Systems Corporation

This statement is required for submission and **will appear in the published article** if the submission is accepted. Please make sure it is accurate.<br><br>**Funded studies**<br>Enter a statement with the following details:<br>• Initials of the authors who received each award<br>• Grant numbers awarded to each author<br>• The full name of each funder<br>• URL of each funder website<br>• Did the sponsors or funders play any role in the study design, data collection and analysis, decision to publish, or preparation of the manuscript?<br><br>Did you receive funding for this work?	
Please add funding details.<br>as follow-up to "**Financial Disclosure**<br><br>Enter a financial disclosure statement that describes the sources of funding for the work included in this submission. Review the submission guidelines for detailed requirements. View published research articles from *PLOS ONE* for specific examples.<br><br>This statement is required for submission and **will appear in the published article** if the submission is accepted. Please make sure it is accurate.<br><br>**Funded studies**<br>Enter a statement with the following details:<br>• Initials of the authors who received each award<br>• Grant numbers awarded to each author<br>• The full name of each funder<br>• URL of each funder website<br>• Did the sponsors or funders play any role in the study design, data collection and analysis, decision to publish, or preparation of the manuscript?	This research was funded by Researchers Supporting Project Number (RSP2024R443), King Saud University, Riyadh, Saudi Arabia.

Did you receive funding for this work?"	
Please select the country of your main research funder (please select carefully as in some cases this is used in fee calculation).<br>as follow-up to "**Financial Disclosure**<br><br>Enter a financial disclosure statement that describes the sources of funding for the work included in this submission. Review the submission guidelines for detailed requirements. View published research articles from *PLOS ONE* for specific examples.<br><br>This statement is required for submission and **will appear in the published article** if the submission is accepted. Please make sure it is accurate.<br><br>**Funded studies**<br>Enter a statement with the following details:<br>• Initials of the authors who received each award<br>• Grant numbers awarded to each author<br>• The full name of each funder<br>• URL of each funder website<br>• Did the sponsors or funders play any role in the study design, data collection and analysis, decision to publish, or preparation of the manuscript?<br><br>Did you receive funding for this work?"	SAUDI ARABIA - SA
**Competing Interests**<br><br>Use the instructions below to enter a competing interest statement for this submission. On behalf of all authors, disclose any competing interests that could be perceived to bias this work—acknowledging all financial support and any other relevant financial or non-financial competing interests.<br><br>This statement is required for submission	The authors have declared that no competing interests exist.

and **will appear in the published article** if the submission is accepted. Please make sure it is accurate and that any funding sources listed in your Funding Information later in the submission form are also declared in your Financial Disclosure statement.<br><br>View published research articles from *PLOS ONE* for specific examples.<br><br>**NO authors have competing interests**<br><br>Enter: *The authors have declared that no competing interests exist.*<br><br>**Authors with competing interests**<br><br>Enter competing interest details beginning with this statement:<br><br>*I have read the journal's policy and the authors of this manuscript have the following competing interests: [insert competing interests here]*<br><br>* typeset	
**Ethics Statement**<br><br>Enter an ethics statement for this submission. This statement is required if the study involved:<br><br>• Human participants<br>• Human specimens or tissue<br>• Vertebrate animals or cephalopods<br>• Vertebrate embryos or tissues<br>• Field research<br><br>Write "N/A" if the submission does not require an ethics statement.<br><br>General guidance is provided below. Consult the submission guidelines for detailed instructions. **Make sure that all information entered here is included in the Methods section of the manuscript.**	N/A

**Format for specific study types**<br><br>**Human Subject Research (involving human participants and/or tissue)**<br>• Give the name of the institutional review board or ethics committee that approved the study<br>• Include the approval number and/or a statement indicating approval of this research<br>• Indicate the form of consent obtained (written/oral) or the reason that consent was not obtained (e.g. the data were analyzed anonymously)<br><br>**Animal Research (involving vertebrate animals, embryos or tissues)**<br>• Provide the name of the Institutional Animal Care and Use Committee (IACUC) or other relevant ethics board that reviewed the study protocol, and indicate whether they approved this research or granted a formal waiver of ethical approval<br>• Include an approval number if one was obtained<br>• If the study involved *non-human primates*, add *additional details* about animal welfare and steps taken to ameliorate suffering<br>• If anesthesia, euthanasia, or any kind of animal sacrifice is part of the study, include briefly which substances and/or methods were applied<br><br>**Field Research**<br><br>Include the following details if this study involves the collection of plant, animal, or other materials from a natural setting:<br>• Field permit number<br>• Name of the institution or relevant body that granted permission	
**Data Availability**<br><br>Authors are required to make all data underlying the findings described fully available, without restriction, and from the time of publication. PLOS allows rare exceptions to address legal and ethical concerns. See the PLOS Data Policy and FAQ for detailed information.	Yes - all data are fully available without restriction

Powered by Editorial Manager® and ProduXion Manager® from Aries Systems Corporation

A Data Availability Statement describing where the data can be found is required at submission. Your answers to this question constitute the Data Availability Statement and **will be published in the article**, if accepted.<br><br>**Important:** Stating 'data available on request from the author' is not sufficient. If your data are only available upon request, select 'No' for the first question and explain your exceptional situation in the text box.<br><br>Do the authors confirm that all data underlying the findings described in their manuscript are fully available without restriction?	
**Describe where the data may be found in full sentences. If you are copying our sample text, replace any instances of XXX with the appropriate details.**<br><br>• If the data are **held or will be held in a public repository**, include URLs, accession numbers or DOIs. If this information will only be available after acceptance, indicate this by ticking the box below. For example: *All XXX files are available from the XXX database (accession number(s) XXX, XXX.).*<br>• If the data are all contained **within the manuscript and/or Supporting Information files**, enter the following: *All relevant data are within the manuscript and its Supporting Information files.*<br>• If neither of these applies but you are able to provide **details of access elsewhere**, with or without limitations, please do so. For example:<br><br>*Data cannot be shared publicly because of [XXX]. Data are available from the XXX Institutional Data Access / Ethics Committee (contact via XXX) for researchers who meet the criteria for access to confidential data.*<br><br>*The data underlying the results presented in the study are available from (include the name of the third party*	All relevant data are within the manuscript and its Supporting Information files.

and contact information or URL).<br>• This text is appropriate if the data are owned by a third party and authors do not have permission to share the data.<br><br>* typeset	
Additional data availability information:	

Households' expenditures for Solid Waste Management Services: influencing factors and deep insight

Ghulam Mustafa[1], Naveed Hayat[1], Bader Alhafi Alotaibi [2*], Abou Traore [3],

[1]Department of Economics, Division of Management and Administrative Science, University of Education, Lahore, Pakistan

[2]Department of Agricultural Extension and Rural Society, College of Food and Agriculture Sciences, King Saud University, Riyadh 11451, Saudi Arabia

[3]Department of Community Sustainability, College of Agriculture and Natural Resources, Michigan State University, 328 Natural Resources Building, East Lansing, MI 48824, USA.
traoreab@msu.edu

*** Correspondence:**
Corresponding Author: Bader Alhafi Alotaibi
balhafi@ksu.edu.sa

Keywords: solid-waste, household, expenditures, waste, management

Abstract

This study aims to document the existing solid waste collection and management system available to households in Pakistan and to explore the factors affecting households' cash payments for waste collection and disposal services. Robust least square regression is applied to household-level data from 16,155 households in the Pakistan Social Living Measurement Survey (PSLM) for 2019-2020. This method was chosen for its ability to handle outliers and provide more reliable estimates. On average, households pay PKR 214 (USD 1.01) per month for waste collection and disposal services. Households in Baluchistan and Khyber Pakhtunkhwa pay the highest amounts, while those in Sindh and Punjab pay less. Rural households pay more than urban households. Waste collection is primarily handled by private vans/carts, with door-to-door collection being the most common method. The municipality's role in waste collection at the doorstep is limited. Public bins are accessible to 83 percent of households, but their distant locations and infrequent emptying pose significant problems. These limitations highlight the need for improved municipal involvement and infrastructure. Results indicate that household income, doorstep waste collection via private van/cart, availability of bins or dumpsters, distance from waste disposal facilities, bin clearance duration, house ownership, dwelling type, and number of rooms significantly affect households' cash payments for waste collection services. To increase cash payments for waste collection services, waste management authorities should provide better and modern solid waste management systems. Upgrading existing systems can enhance households' willingness to pay for these services.

1 Introduction

Due to the rapid growth of economic, urbanization, industrialization and population in urban areas, the demand for solid waste management has increased significantly (Adeleke et al., 2022). Globally, the annual generation of solid waste exceeds 2 billion tonnes (Kaza and Lisa, 2018) and this is expected to increase to 2.2 billion tonnes in 2025 (Hoornweg and Bhada-Tata, 2012). The situation would be further exacerbated with the population of about 9.7 billion by 2050 (Dastjerdi et

al., 2021) with estimated solid waste of 3.40 billion tonnes (Kaza et al., 2018). Presently, only about 38% of solid waste is being managed in an environmentally friendly manner, while the rest is handle in an unsustainable way, resulting in environmental challenges such as ecosystem damage, abiotic resources depletion, human health hazards, ozone depletion, and global warming (Dastjerdi et al., 2021; Khandelwal et al., 2019). Currently, cost of solid waste management is $205.5 billion which is expected to increase $375.5 billion in 2025 (Hoornweg and Bhada-Tata, 2012). In developing countries, about 30 percent to 50 percent of municipal operation budget spent on collection of waste and its management (Afroz et al., 2011; Rahji and Oloruntoba, 2009). Similarly, households also spend a huge cash payment for solid waste management which is still undocumented yet. Therefore, this study is designed to find households cash payment for solid waste management.

The amount of per-capita waste generation ranges 0.11 kg to 4.54kg with average of 0.74kg per person per day (Abarca-Guerrero et al., 2015). Usually, wastes are managed by incineration, landfills, dumps and composting processes. The increase in solid waste have many environmental repercussions such as greenhouse gas emission and devastating pollution. Unmanageable solid waste accumulation deteriorates human health, affects landscapes, spread diseases, and harms the urban environment (Khair et al., 2019; Vassanadumrongdee and Kittipongvises, 2018). For instance, it has been estimated that 3.3 billion tonnes of CO2 is accumulating into the atmosphere every year due to food waste which is the major portion of municipal solid waste (Pattnaik and Reddy, 2010; Tayyab et al., 2019). Even in case of recycling of solid waste, it is possible of potential health impacts through recuperation in circular economy supply chain (Kaza et al., 2018).

There are number of factors that affect the households' cash payment to collect solid waste. Among others, income of household head is the major determinant of cash payment of solid waste management. The studies have empirically proved that financially well-off families has positive attitude toward waste management and hence their propensity to pay for waste disposal is higher (Omotayo et al., 2020; Oyekale, 2015). Tadesse et al. (2007) were of the opinion that families with higher incomes use communal waste containers and they are less likely to dispose of waste on unapproved places. Therefore, income can be considered strong predictor of cash payment of solid waste disposal.

Access to solid waste management services significantly impact the household cash payment to manage the waste. Endalew and Tassie (2018) were of the opinion that access to waste management services create environmental awareness and their negative impacts on health among the respondents. Therefore, such services motivate the households to pay for waste collection services. Similarly, Chukwuone et al. (2022) found that availability of dumpsters in the locality significantly reduce the likelihood of illegal waste disposal. If waste management services are not provided, it can create isolation among individual and create fear of liability (Bennets, 2008). Hence, it can be concluded that users of solid waste management services pay higher amount for waste management and it is one of the major factors affecting cash payment for waste management services.

Usually people reluctant to pay for waste disposal and its management. However, they willing to spare their time for such services including cleanup activities, involvement in voluntary service and membership of an association. It can be measured in man-days or hours worked in cleaning or disposal activities. For instance, Chukwuone et al. (2022) estimated that the number of man-days or hours they were willing to participate in a week on waste disposal activities. They found that man-days in cleaning activities significantly and positively impact the waste management. Most of times, households give time instead of cash payment: economists refer this as opportunity cost. In this case, their willingness to pay cash decreases due to involvement in cleaning or disposal of waste activities.

Pakistan generates 30 million metric tons of municipal solid waste per year, which has been increasing at the rate of 2.4 percent annually. Moreover, a substantial increase in solid waste in the coming years is expected due to rapid population growth, urbanization, and economic development in the country. However, like other developing countries, Pakistan lacks proper waste management infrastructure which creating serious environmental problems in the country. Most municipal waste is either burned, dumped, or buried on vacant lots, threatening the health and welfare of the common masses. The country facing enormous challenges on how to manage solid waste. Bureaucratic hurdles, lack of urban planning, inadequate waste management equipment, and low public awareness contribute to the problem. Therefore, Pakistan urgently needs a waste road map for its policy makers, to make progress toward better health for its residents and reduce the contamination of land (ADB, 2022). Due to the importance of the issue under consideration, this study aims to document the existing solid waste collection and management system available to households in Pakistan. The study also explores the factors affecting the households' cash payments for waste collection and disposal services. Findings of the study will provide help the policymakers in devising proper waste management policies at national, provincial, districts and tehsil level to make the existing solid waste collection and management system more effective and to make progress toward better health for its people.

2 Manuscript Formatting

2.1 Data

This study used the recent data of the Pakistan Social Living Measurement Survey (PSLM) for the year 2019-2020, conducted by the Pakistan Bureau of Statistics. From this data we select data of 16,155 households who pay cash for waste collection and disposal services. This dataset provides information on the households' monthly cash payment for waste collection and disposal services, households monthly income, households waste management techniques, average time the households spent on a round trip to the nearest public bin/collection point, duration of the nearest public bin emptied/cleared, dwelling type of households, region, and province to which the households belongs.

2.2 Empirical Methods

The empirical model we employ for this study is based on a utility maximization problem proposed by Omotayo et al. (2020) and Oyekale (2015) . The utility maximization problem is used to derive the function for the empirical modelling of household decisions regarding the disposal of solid wastes through cash payments. Assume an economy of consisted of N identical households with utility functions represented by:

$$u_i = u(x_i, e_i) \quad (1)$$

where x_i is the composite goods and services consumed by ith household and e_i is the environmental quality that is being enjoyed by ith household. Suppose the environmental quality function is expressed as:

$$e_i = e(g_i, h_i) \quad (2)$$

where g_i is the quantity of solid wastes that was disposed by ith household. The values of g_i in the environmental quality model e_i is related to a set of some socio-demographic characteristics h_i possessed by the households. If equation (2) is directly substituted into equation (1), the final expression of consumer's utility function is derived as:

$$u_i = u[x_i, e(g_i, h_i)] \quad (3)$$

This utility function given in equation (3) can be maximized subject to the budget constraint faced by the ith household. The expression of budget constraint is presented as:

$$x_i + p_g g_i = m_i \quad (4)$$

It should be noted that in equation (4), the price of x_i is normalized to unity, p_g is unit price of waste disposal, and m_i represents total income of ith household. Solving the utility maximization problem, we get the following waste disposal function:

$$g_i = g(p_g, m_i, h_i) \quad (5)$$

From available data, payment for waste disposal g_i is associated with equation (5), although the dependent variable is continuous in nature (household monthly cash payment for waste collection and disposal services in PKR). The nature of the dependent variable naturally suggests estimation of liner regression to determine the factors affecting waste disposal through cash payments. Therefore, we devise the following regression model:

$$g_i = \alpha + \beta m_i + \gamma_{ik} h_i + \varepsilon_i \quad (6)$$

where α, β, and γ_i are the parameters to be estimated and ε_i is the error term. In our model the dependent variable is household monthly cash payment for waste collection and disposal services (Chukwuone et al., 2022), the explanatory variables include: household monthly income (Ekperi et al., 2019; Endalew & Tassie, 2018; Omotayo et al., 2020: Oyekale, 2015; Pan et al., 2022; Tassie & Endalew, 2020), household waste management techniques (Chukwuone et al., 2022; Endalew & Tassie, 2018; Tassie & Endalew, 2020), average time the household spent on a round trip to the nearest public bin/collection point (Ahmed, 2022; Mulat et al., 2019), duration of the nearest public bin emptied/cleared (Ahmed, 2022; Mulat et al., 2019), dwelling type (Adzawla et al., 2019; Ahmed, 2022), regional dummies (Adzawla et al., 2019), and provincial (Adzawla et al., 2019) dummies as explained in Table 1 (in next section).

3 Results and Discussion

3.1 Existing solid waste collection and management system available to households

For accomplishing the study objectives, we use the data of 16155 households from Pakistan Social and Living Measurement Survey (PSLM) for the year 2019-20. Figure 1 shows that out of 16155 households, 59.15% (9555) households belong to Punjab, 34.61% (5592) households belong to Sindh, 4.54% (733) households belong to Baluchistan, and only 1.70% (275) households belong to Khyber-Pakhtunkhwa. Similarly, out of 16155 households, 89.79% (14505) households living in urban areas whereas 10.21% (1650) household living in rural areas of the country.

Figure 1: Number of households selected for the study

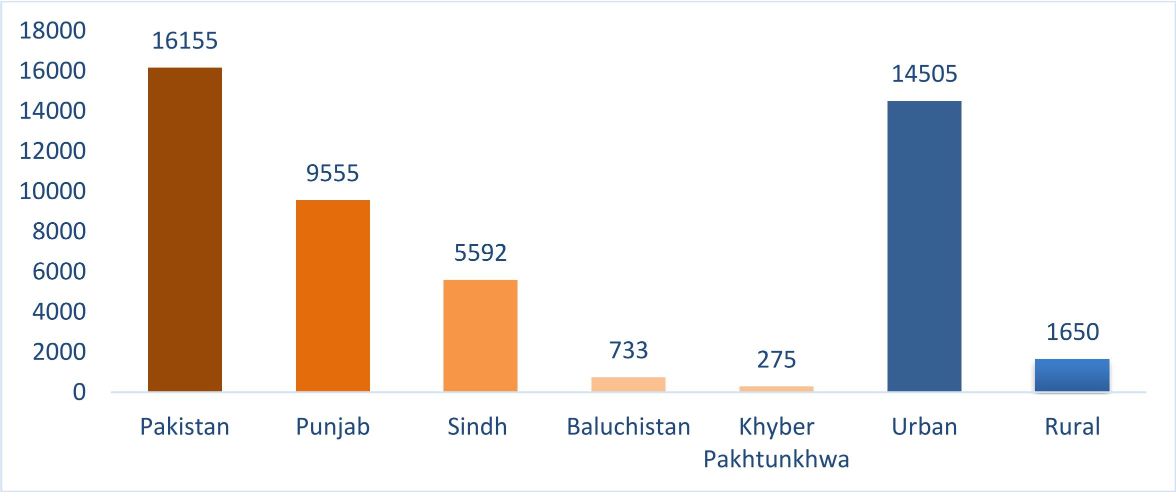

Source: Estimated by authors based on PSLM data for the year 2019-20.

Punjab is most populous province and that is why sample size is large however, Baluchistan is biggest in area but least populous province. Conversely, households from Baluchistan on average pay the highest money of PKR 382 per month for waste collection and disposal services, followed by the households in Khyber Pakhtunkhwa (PKR 263 per month), households in Sindh (PKR 228 per month), and households in Punjab (PKR 191 per month) as shown in Figure 2. Similarly, from Figure 2, it is observed that the rural household pay more cash (PKR 308 per month) for waste collection and disposal services as compared to the urban households (PKR 203 per month). Furthermore, from Figure 2 we observe that in Pakistan on average the households pay PKR 214 (Approximately USD 1; 1USD=PKR 220) per month for waste collection and disposal services. Current solid waste management system in the country is poor and needs more improvement. The study has found that people willingness to pay regarding waste management is USD 4.8 in Pakistan (Akhtar et al., 2017). Therefore, study recommends that for increasing the cash payment for waste collection and disposal services the waste collection authorities must provide better and modern solid waste management system to the households. The upgradation of the existing solid waste management system can also increase the households cash payments for waste collection and disposal services.

Figure 2: Household cash payment for waste collection and disposal services (PKR/Month)

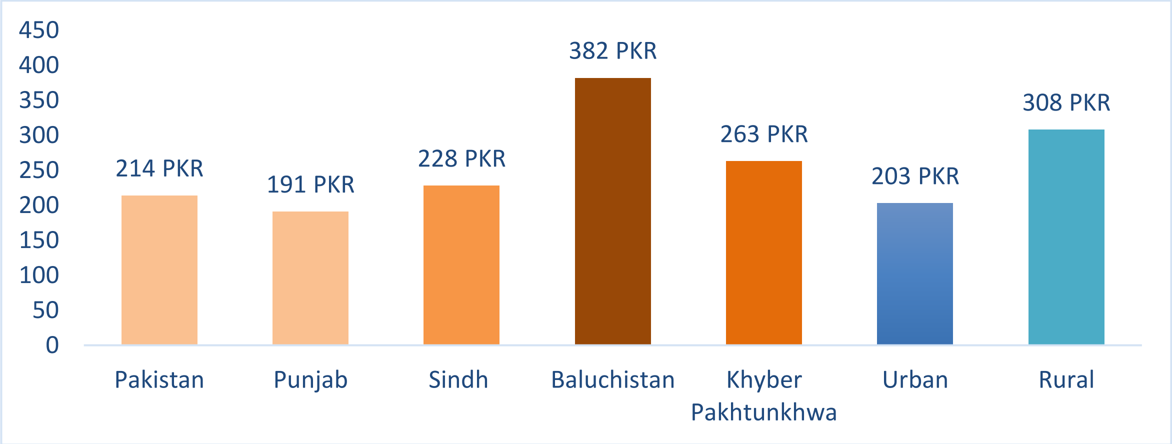

Source: Estimated by authors based on PSLM data for the year 2019-20.

Form Figure 3 we observed that in Pakistan the average monthly household income is PKR 25497. Households from Baluchistan have the highest average monthly income of PKR 28614, followed by

the households in Khyber Pakhtunkhwa (PKR 28356), households in Punjab (PKR 25716), and households in Sindh (PKR 24574). Similarly, the monthly income of the rural household is higher (PKR 27546) than that of the monthly income of the urban households (PKR 25264). The results can have two compensations. Firstly, wealthier people pay more cash payment for solid waste management. For instance, Baluchistan has higher average income and thus pays more for solid waste management system. Similarly, rural residents have higher mean income and thus they spend more for garbage collection as compared to urban counterparts. This might be the reason that there is well established system of waste management in urban areas provided by government and hence urban residents spend less money for waste collection. On the other hands, waste collection infrastructure is very poor in rural areas and therefore rural people spend more money for waste disposal. Secondly, waste management is need rather than choice and people spend money on it despite to their financially soundness. For instance, Punjab province has lower mean income than Sind but still former pays more than later.

Figure 3: Household income (PKR/Month)

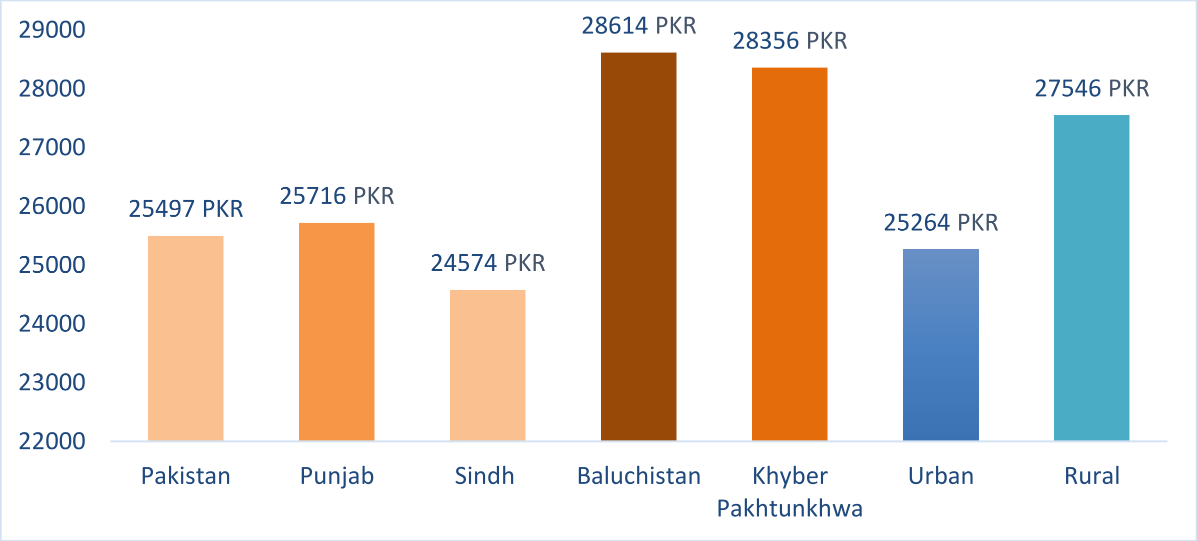

Source: Estimated by authors based on PSLM data for the year 2019-20.

From Figure 4, it is observed that out of 16155 households, waste from 72 percent of households has been collected by private van/cart from door step whereas waste from 28 percent of households has been collected by municipality van from door step. This show the municipality role in collecting the waste from the households is still limited in the country. The municipal corporations in the big cities are under the control of city government whereas in small town and rural setup it works under the tehsil governments. However, in the country the local government system is not so powerful, thus the municipalities get a small budget and resultantly they are unable to reach to every corner of the city or to each rural area. Thus, they are collecting a small portion of waste from the households and the remaining households are bound to dispose their waste using private van or carts.

Figure 4: Households waste collection

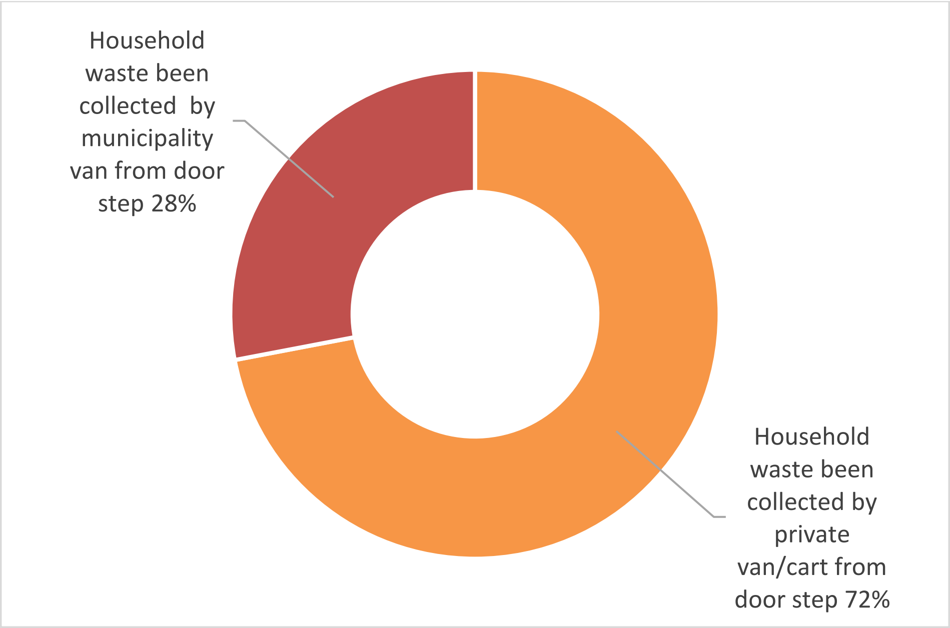

Source: Estimated by authors based on PSLM data for the year 2019-20.

From Figure 5 we observe that for waste collection and disposal bin is available/accessible to 83 percent of the households whereas bin is not available/accessible to 17 percent of the households.

Figure 5: Bin availability/accessibility to households

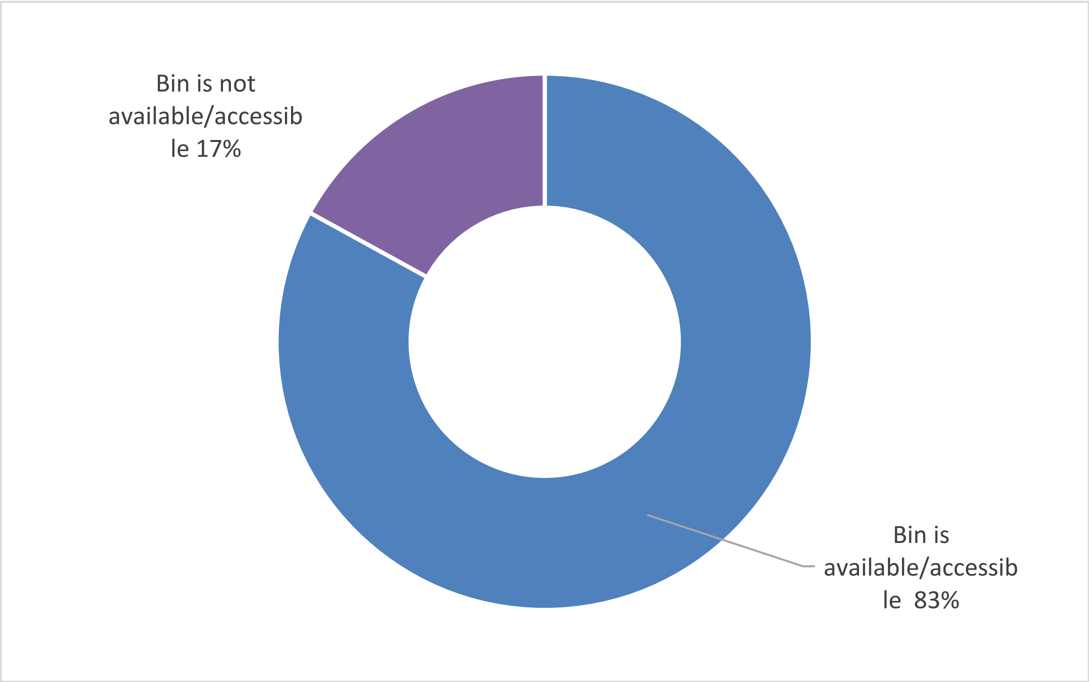

Source: Estimated by authors based on PSLM data for the year 2019-20.

Figure 6 shows that the 52 percent of the household spent 1 to 5 minutes on a round trip to the nearest public bin/collection point. Similarly, 19 percent of the household spent 6 to 10 minutes, 8 percent of the households spent 11 to 15 minutes, 3 percent of the households spent 16 to 20 minutes, 1 percent of the households spent 21 to 25 minutes, and 1 percent of the household spent 26 and more minutes on a round trip to the nearest public bin/collection point, respectively.

Figure 6: Average time the household spent on a round trip to the nearest public bin/collection point

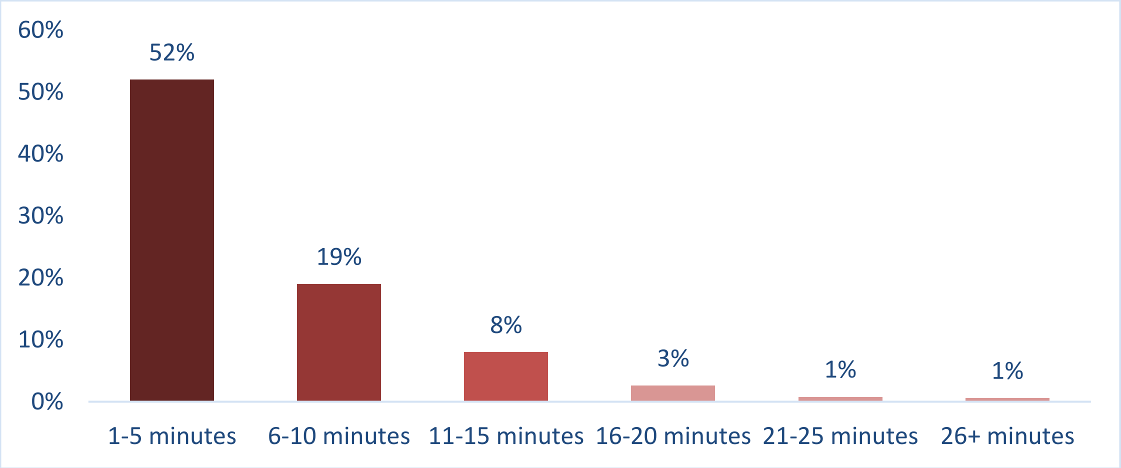

Source: Estimated by authors based on PSLM data for the year 2019-20.

In Figure 7, 42 percent of the households reported that the nearest public bin available to them is emptied/cleared by the concern person(s) or local authority(s) every day. In similar fashion, 11 percent of the households reported this duration once a week, 6 percent of the households reported this duration twice a week, and only 3 percent of household reported this duration thrice a week, respectively. On the other hand, 38 percent of the household don't know about the nearest public bin emptied duration. This is a good sign that in the country for waste disposal bin is available to majority of households. However, the real problem is that these bins are far away from the house site and the households spent more time to reach to bin for waste disposing. Therefore, it is recommended that the authorities put the bin near to the households' residence that they are dispose their waste in lesser time and lesser efforts. The second problem, is these bins are emptied/cleared by the concern person(s) or local authority(s) after few days. If the bin is not emptied on time than it can cause various diseases and can adversely affect the households' health. Therefore, it is recommended that the authorities must devise proper and quick method for clearing the bin in the recommended time period.

Figure 7: Duration of the nearest public bin emptied/cleared

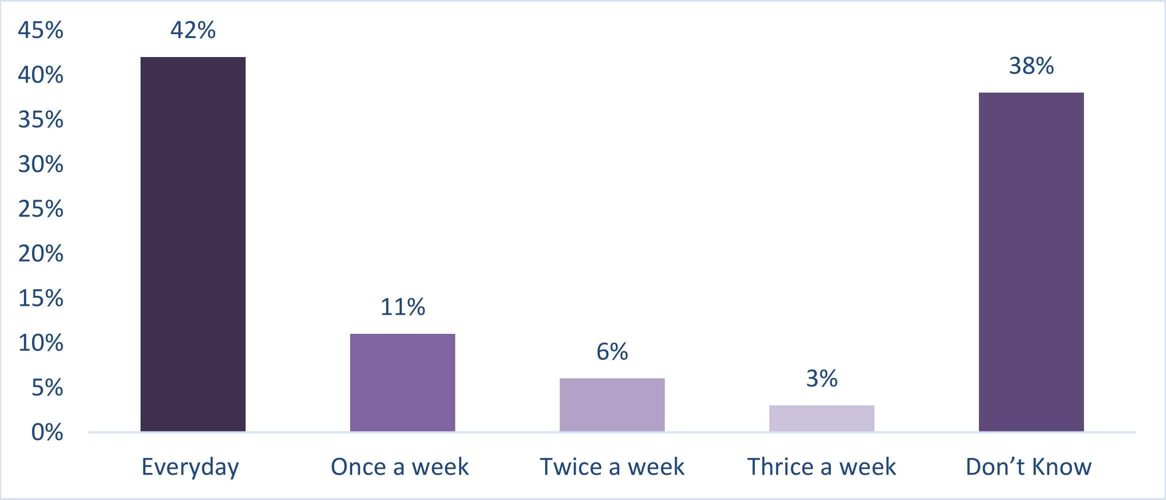

Source: Estimated by authors based on PSLM data for the year 2019-20.

3.2 Descriptive Statistics

Descriptive statistics and the definition of the important variables used in this study is given in Table 1. It is observed that on average the Pakistani households pay PKR 214 per month for waste collection and disposal services. Contrasting the explanatory variables, we find that the average monthly household income in Pakistan is PKR 25497. Comparing the Household waste management characteristics, it is observed that from 72 percent of households' waste has been collected by private van/cart from door step. For waste collection and disposal bin is available/accessible to 83 percent of the households. Furthermore, 52 percent of the household spent 1 to 5 minutes on a round trip to the nearest public bin/collection point. Similarly, 19 percent of the household spent 6 to 10 minutes, 8 percent of the households spent 11 to 15 minutes, 3 percent of the households spent 16 to 20 minutes, only 1 percent of the households spent 21 to 25 minutes on a round trip to the nearest public bin/collection point, respectively. 42 percent of the households reported that the nearest public bin available to them is emptied/cleared by the concern person(s) or local authority(s) every day. In similar fashion, 11 percent of the households reported this duration once a week, 6 percent of the households reported this duration twice a week, and only 3 percent of household reported this duration thrice a week, respectively. Comparing the household housing characteristics, it is observed that 83 percent of the households living in their own houses whereas 10 percent of the households living in rented houses. Moreover, the households are living on average in 2 rooms houses. Similarly, 77 percent of the households living in independent houses, 13 percent of the households living in part of large units, 7 percent of the households living in part of compounds, and only 3 percent of households living in apartments. Furthermore, 90 percent of the household living in urban areas. Finally, 59 percent of the households living in Punjab, 36 percent of the households living in Sindh, and 4.5 living in Baluchistan.

Table 1: Descriptive Statistics

Variables	Definition	Mean	Standard Deviation
Dependent			
Household cash payment for waste collection and disposal services	Household monthly cash payment for waste collection and disposal services (PKR)	214	277
Explanatory			
Household income:			
Income	Household monthly income (PKR)	25497	53289
Household waste management techniques:			
Household waste been collected by private van/cart from door step	1 if the household waste been collected by private van/cart from door step, 0 otherwise	0.72	0.45
Bin available/accessible	1 if bin is available/accessible to household, 0 otherwise	0.83	0.38
Average time the household spent on a round trip to the nearest public bin/collection point:			
1 – 5 minutes	1 if the household spent 1 to 5 minutes on a round trip to the nearest public bin/collection point, 0 otherwise	0.52	0.50
6 – 10 minutes	1 if the household spent 6 to 10 minutes on a round trip to the nearest public bin/collection point, 0 otherwise	0.19	0.39
11 – 15 minutes	1 if the household spent 11 to 15 minutes on a round trip to the nearest public bin/collection point, 0 otherwise	0.8	0.27

16 – 20 minutes	1 if the household spent 16 to 20 minutes on a round trip to the nearest public bin/collection point, 0 otherwise	0.3	0.16
21 – 25 minutes	1 if the household spent 21 to 25 minutes on a round trip to the nearest public bin/collection point, 0 otherwise	0.1	0.09
Duration of the nearest public bin emptied/cleared:			
Every day	1 if the nearest public bin is emptied/cleared every day, 0 otherwise	0.42	0.49
Once a week	1 if the nearest public bin is emptied/cleared once a week, 0 otherwise	0.11	0.32
Twice a week	1 if the nearest public bin is emptied/cleared twice a week, 0 otherwise	0.6	0.24
Thrice a week	1 if the nearest public bin is emptied/cleared thrice a week, 0 otherwise	0.3	0.17
Household housing characteristics:			
Own house	1 if the household living in his own house, 0 otherwise	0.83	0.38
On rent	1 if the household living in a rented house, 0 otherwise	0.10	0.31
Number of rooms in the house	Number of rooms in the house	2.3	1.3
Dwelling type:			
Independent house	1 if the household live in independent house, 0 otherwise	0.77	0.42
Apartment	1 if the household live in apartment, 0 otherwise	0.03	0.18
Part of the large unit	1 if the household live in part of a large unit, 0 otherwise	0.13	0.39
Part of compound	1 if the household live in part of a compound, 0 otherwise	0.07	0.25
Region:			
Urban	1 if the household belongs to urban area, 0 otherwise	0.90	0.30
Province:			
Punjab	1 if the household belong to Punjab, 0 otherwise	0.59	0.49
Sindh	1 if the household belong to Sindh, 0 otherwise	0.36	0.48
Baluchistan	1 if the household belong to Baluchistan, 0 otherwise	0.045	0.13
Observations	Number of households	16155	

Source: Estimated by authors based on PSLM data for the year 2019-20.

3.3 Factors affecting the households cash payments for waste collection and disposal services

The results of the estimated regression for Pakistan are given in Table 2, while the results of the estimated regressions for the four provinces are given in Table 3 (Punjab, Sindh, Khyber Pakhtunkhwa, Baluchistan). The results of the diagnostic check (R squared, F statistics and Root MSE) of all the regressions are reported in the last panel of Table 2 and Table 3, respectively. In our seven regression models, the R squared is in the range of 0.04 to 0.28, while root mean square error (Root MSE) is in the range of 0.52 to 0.71. Results of the F statistics show that the regression models are overall statistically significant at 1 and 5 percent level for each of the seven regressions.

Table 2: Results of robust least square regression for Pakistan

Model:	1	2	3
	Pakistan	Urban	Rural
Dependent variable: Household cash payment for waste collection and disposal services (ln)			
Household income:			
Income (ln)	-0.00607	-0.00525	-0.0208
	(0.00593)	(0.00600)	(0.0236)
Household waste management techniques:			
Household waste been collected by municipality van from door step (Reference)			
Household waste been collected by private van/cart from door step (Yes = 1, No = 0)	0.0857***	0.0948***	-0.225***
	(0.00563)	(0.00546)	(0.0495)
Bin available/accessible (Yes = 1, No = 0)	-0.0767*	-0.0885*	-0.140
	(0.0460)	(0.0452)	(0.231)
Average time the household spent on a round trip to the nearest public bin/collection point:			
26+ minutes (Reference)			
1 – 5 minutes (Yes = 1, No = 0)	0.173***	0.133***	0.576**
	(0.0462)	(0.0453)	(0.230)
6 – 10 minutes (Yes = 1, No = 0)	0.210***	0.181***	0.476**
	(0.0463)	(0.0456)	(0.232)
11 – 15 minutes (Yes = 1, No = 0)	0.221***	0.196***	0.396
	(0.0481)	(0.0475)	(0.251)
16 – 20 minutes (Yes = 1, No = 0)	0.214***	0.195***	0.175
	(0.0540)	(0.0538)	(0.268)
21 – 25 minutes (Yes = 1, No = 0)	0.171***	0.141**	0.296
	(0.0632)	(0.0629)	(0.253)
Duration of the nearest public bin emptied/cleared:			
Don't Know (Reference)			
Every day (Yes = 1, No = 0)	-0.0380***	-0.0223*	-0.162***
	(0.0135)	(0.0135)	(0.0558)
Once a week (Yes = 1, No = 0)	-0.0293*	0.000959	-0.374***
	(0.0174)	(0.0170)	(0.0801)
Twice a week (Yes = 1, No = 0)	0.0109	0.0522***	-0.328***
	(0.0198)	(0.0201)	(0.0773)
Thrice a week (Yes = 1, No = 0)	0.180***	0.225***	-0.164
	(0.0379)	(0.0393)	(0.137)
Household housing characteristics:			
Rent free (Reference)			
Own house (Yes = 1, No = 0)	0.0327*	0.0427**	-0.0700
	(0.0178)	(0.0169)	(0.0863)
On rent (Yes = 1, No = 0)	-0.0111	-0.00451	-0.0608
	(0.0220)	(0.0212)	(0.0988)
Number of rooms in the house	-0.00305	0.000255	-0.0224*
Dwelling type:			
Other dwelling type (Reference)			
	(0.00339)	(0.00342)	(0.0134)
Independent house (Yes = 1, No = 0)	-0.147**	-0.120**	-0.411
	(0.0660)	(0.0565)	(0.352)

Apartment (Yes = 1, No = 0)	-0.201***	-0.183***	-0.265
	(0.0704)	(0.0612)	(0.389)
Part of the large unit (Yes = 1, No = 0)	-0.132**	-0.119**	-0.316
	(0.0669)	(0.0574)	(0.354)
Part of compound (Yes = 1, No = 0)	-0.167**	-0.137**	-0.406
	(0.0674)	(0.0582)	(0.357)
Region:			
Rural (Reference)			
Urban (Yes = 1, No = 0)	-0.0817***		
	(0.00958)		
Province:			
Khyber Pakhtunkhwa (Reference)			
Punjab (Yes = 1, No = 0)	-0.361***	-0.352***	-0.330***
	(0.0401)	(0.0433)	(0.0843)
Sindh (Yes = 1, No = 0)	-0.159***	-0.150***	-0.891***
	(0.0404)	(0.0435)	(0.113)
Baluchistan (Yes = 1, No = 0)	-0.0183	-0.0306***	0.0143
	(0.0121)	(0.0117)	(0.0264)
Constant	5.536***	5.328***	6.504***
	(0.0993)	(0.0936)	(0.452)
Diagnostic check:			
Observations	16,155	14,505	1,650
R-squared	0.077	0.082	0.122
F Statistics	53.10***	57.86***	8.18***
Root MSE	0.566	0.541	0.716

Source: Estimated by authors based on PSLM data for the year 2019-20. Note: Robust standard errors in parentheses, *** p<0.01, ** p<0.05, * p<0.1.

From Table 2 (Pakistan, rural and urban), we found statically insignificant impact of income on the households' cash payment for solid waste collection and disposal services. These results go in line with a priori expectation given that prevailing differences in the attitudes of households towards waste collection and disposal services are often borne out of their income and poverty levels. In the developing country like Pakistan, households are mostly poor and unable to independently support their living expenses. Therefore, when their income increases they increase their expenses on other essentials like food items, fuel, utilities, footwear, clothing, housing, education, and health while the increase in their incomes cannot affect their payment on waste collection and disposal. For Khyber Pakhtunkhwa and Baluchistan (Table 3), we find similar results. However, in Punjab (Table 3) we observed that an increase in household income increase their cash payment for solid waste collection and disposal services whereas in Sindh an increase in household income brings a significant decrease in their cash payments for waste collection and disposal services. Punjab is the largest province of Pakistan by population, the province has relatively developed agriculture, industrial and services sector as compared to other provinces. Therefore, the households in this province give proper attention to waste management and hence their cash payment for waste collection and disposal increases as their income increases. Conversely, by population Sindh is the second largest province of the country, the province is poor as compared to the Punjab. Therefore, any increase in the households' income decrease their payment on waste disposal. In related studies, Oyekale (2015) and Omotayo et al. (2020) found that household's income positively affecting their payment for waste disposal. In other studies, Endalew & Tassie (2018). Ekperi et al. (2019), Mulat et al. (2019), and

Tassie & Endalew (2020) found that households income positively affect their willingness to pay for solid waste management system and services.

Table 3: Results of robust least square regression for four provinces of Pakistan

Model:	4	5	6	7
Province:	Punjab	Sindh	Baluchistan	Khyber Pakhtunkhwa
Dependent variable: Household cash payment for waste collection and disposal services (ln)				
Household income:				
Income (ln)	0.0131*	-0.0335***	-0.0126	0.0654
	(0.00786)	(0.00942)	(0.0296)	(0.0490)
Household solid waste management techniques:				
Household waste been collected by municipality van from door step (Reference)				
Household waste been collected by private van/cart from door step (Yes = 1, No = 0)	0.0946***	0.0879***	0.0991**	-0.105**
	(0.00703)	(0.0106)	(0.0481)	(0.0462)
Bin available/accessible (Yes = 1, No = 0)	-0.00694	-0.154***	0.682***	-1.930***
	(0.0637)	(0.0560)	(0.115)	(0.187)
Average time the household spent on a round trip to the nearest public bin/collection point:				
26+ minutes (Reference)				
1 – 5 minutes (Yes = 1, No = 0)	0.0298	0.206***	0.417***	1.695***
	(0.0635)	(0.0562)	(0.105)	(0.117)
6 – 10 minutes (Yes = 1, No = 0)	0.0341	0.350***	0.0357	1.531***
	(0.0641)	(0.0565)	(0.106)	(0.141)
11 – 15 minutes (Yes = 1, No = 0)	0.123*	0.297***	-0.168	1.911***
	(0.0668)	(0.0604)	(0.115)	(0.191)
16 – 20 minutes (Yes = 1, No = 0)	0.171**	0.110	0.0460	2.019***
	(0.0731)	(0.0729)	(0.157)	(0.216)
21 – 25 minutes (Yes = 1, No = 0)	0.0666	0.178	0.106	1.633***
	(0.0793)	(0.111)	(0.157)	(0.159)
Duration of the nearest public bin emptied/cleared:				
Don't Know (Reference)				
Every day (Yes = 1, No = 0)	-0.0158	0.0508**	-0.640***	-0.215**
	(0.0173)	(0.0211)	(0.100)	(0.0940)
Once a week (Yes = 1, No = 0)	0.00670	-0.0110	-0.682***	0.200
	(0.0268)	(0.0228)	(0.0837)	(0.123)
Twice a week (Yes = 1, No = 0)	0.0519**	-0.00234	-0.327***	-0.238*
	(0.0248)	(0.0349)	(0.0983)	(0.131)
Thrice a week (Yes = 1, No = 0)	0.270***	0.0343	-0.314**	0.128
	(0.0465)	(0.0744)	(0.139)	(0.278)
Household housing characteristics:				
Rent free (Reference)				
Own house (Yes = 1, No = 0)	0.0596**	0.00734	-0.101	-0.124
	(0.0236)	(0.0267)	(0.113)	(0.121)
On rent (Yes = 1, No = 0)	0.0416	-0.0666**	-0.247*	-0.118
	(0.0289)	(0.0330)	(0.135)	(0.174)

Number of rooms in the house	0.00440	-0.0119**	-0.00646	0.0603*
	(0.00465)	(0.00505)	(0.0186)	(0.0358)
Dwelling type:				
Other dwelling type (Reference)				
	-0.111	-0.169*	0.0369	-0.675***
Independent house (Yes = 1, No = 0)	(0.0891)	(0.0929)	(0.160)	(0.167)
	-0.237**	-0.0900		-0.287*
Apartment (Yes = 1, No = 0)	(0.0948)	(0.0984)		(0.159)
	-0.0800	-0.227**	0.195	-0.320*
Part of the large unit (Yes = 1, No = 0)	(0.0899)	(0.0947)	(0.166)	(0.184)
	-0.129	-0.188*	0.181	-1.024***
Part of compound (Yes = 1, No = 0)	(0.0906)	(0.0962)	(0.194)	(0.233)
Region:				
Rural (Reference)				
Urban (Yes = 1, No = 0)	-0.0764***	0.0902***	-0.0179	-0.193***
	(0.0104)	(0.0242)	(0.0303)	(0.0513)
Constant	4.924***	5.355***	5.045***	6.004***
	(0.122)	(0.147)	(0.373)	(0.581)
Diagnostic check:				
Observations	9,555	5,592	733	275
R-squared	0.050	0.042	0.289	0.258
F Statistics	22.60***	13.85***	10.78***	5.28**
Root MSE	0.564	0.527	0.661	0.577

Source: Estimated by authors based on PSLM data for the year 2019-20. Note: Robust standard errors in parentheses, *** p<0.01, ** p<0.05, * p<0.1.

Mostly household in Pakistan pay for the waste collection from door. This exactly can be seen that if van/cart collect the waste from door, households' cash payment for waste collection and disposal increase both positively and significantly. This is true in case of Pakistan and urban areas, respectively. However, in rural areas if van/cart collect the garbage from door, households cash payment decreases. It has two compensations; firstly, households in rural areas are poor, and secondly, large proportion of garbage of rural households consisted of animal dung and people prefer to throw in their agricultural field rather than to give to waste disposal cart/van. Similarly, van/cart services positively and significantly impact the households' cash payment for waste disposal in Punjab, Sindh and Baluchistan provinces, respectively (Table 3). However, in Khyber Pakhtunkhwa van/cart services for waste collection significantly and negatively impact the cash payment. In the other studies, Endalew & Tassie (2018) and Tassie & Endalew (2020) linked the waste collection from door with households' willingness to pay solid waste management system and services. They found that that access to solid waste management services positively affect the households' willingness to pay solid waste management system and services.

Infrastructure availability is most important factor affecting the households' cash payment for waste disposal. If infrastructure availability is present it reduces households' efforts and cash. For instance, if the availability of bin or dumpster is there in the nearby locality, households' cash payment significantly reduces in Pakistan (Table 2). Same is true for urban area where cash payment reduces, however, infrastructure availability such as bins or dumpsters are not enough to capture any meaningful impact of it on rural households' cash payment of waste management. Table 3 depicts

that bin/dumpster availability negatively impact the cash payment in Sindh and Khyber Pakhtunkhwa (KPK). It means that access on public bin/dumpster lead households to pay cash for waste disposal. On other hand, in Sindh, access on public bin negatively impact the cash payment by households. This is the reason people throw waste in/around the public bin and that is why waste management condition is worst in Sindh (reference). In other studies, Chukwuone et al. (2022) linked the availability of dumpster with illegal waste disposal and found that the availability of dumpster in a residential site significantly reduce the likelihood of illegal waste disposal.

Average time the household spent on a round trip to the nearest public bin/collection point significantly affect the households cash payment for waste collection and disposal services in Pakistan. However, when the average time to the nearest public bin increases the households cash payment increase reached to a certain threshold (11 – 15 minutes) then decreasing again when the average time to the nearest public bin increases further. The same tendency is observed for urban areas. This shows that if the waste disposal facility sites are situated near to the residential sites such that on the distance of 1 – 15 minutes, the households cash payment for waste collection and disposal services increase. On the other hand, if the distance from solid waste disposal facility increases the households cash payment gradually falls. This is because if the longer the distance the more complicate problem of waste collection and disposal as households would have to walk a long distance to dispose their waste. Conversely, in rural areas when the average time to the nearest public bin increases from 1 – 5 minutes to 6 – 10 minutes the households cash payment for waste collection and disposal services decrease. Since most of the rural households are poor and they prefer to throw their waste (mostly animal dung) in their agricultural fields rather than to dispose it in bin, therefore, it is not matter for them that the bin is near to their house or far away from their residential site. In Punjab the households cash payment for waste collection and disposal services increases when the average time to the nearest public bin increases from 11 – 15 minutes to 16 – 20 minutes. On the other hand, in Sindh, when the average time to the nearest public bin increases from 1 – 5 minutes to 6 – 10 minutes the households cash payment for waste collection and disposal services decrease. In Baluchistan the household cash payment for waste collection and disposal services increase if and only if the bin is near to the housing site (1 – 5 minutes). In Khyber Pakhtunkhwa when the average time to the nearest public bin increases from 1 – 5 minutes to 6 – 10 minutes the households cash payment for waste collection and disposal services decrease. However, the cash payment increases again when the average time to the nearest public bin increases from 11 – 15 minutes to 16 – 20 minutes. Other studies like Mulat et al. (2019) and Ahmed (2022) linked the distance between resident area and waste disposal facility with households willingness to pay for improved solid waste management services and found a significant positive effect of this variable on the household willingness to pay for improved solid waste management services.

In Pakistan, if the nearest public bin available to households is emptied/cleared by the concern person(s) or local authority(s) every day or twice a week then households are pay less cash for waste collection and disposal. However, if the bin is cleared thrice a week then households are pay more for waste collection and disposal services. In urban areas of the country, if the nearest public bin is emptied every day then households are pay less cash for waste collection and disposal. However, if the bin is cleared twice a week or thrice a week then households are pay more for waste collection and disposal services. This shows that if the bins are emptied by the concern person(s) or local authority(s) after few days then households cash payment for waste disposal increases. It is reasonable because, if the bin is not emptied on time than it can cause various diseases and can adversely affect the households' health. In order to avoid the health problem, the households whose bins are cleared after few days such that twice or thrice a week are pay more cash for waste disposal services. On the other hand, in rural areas of the country, the households cash payment for waste

collection and disposal decreases regardless of the duration of the bin cleared. The duration of the public bin clearance is not the matter of rural households because most of the rural households prefer to throw their waste (mostly animal dung) in their agricultural fields rather than to dispose it in public bin. In Punjab, if the bin is cleared twice or thrice a week then households are willing to pay more cash for waste collection and disposal services. In Sindh, if the nearest public bin is emptied every day then households are pay more cash for waste collection and disposal. However, in Baluchistan and Khyber Pakhtunkhwa, the households cash payment for waste collection and disposal services decreases regardless of the duration of the bin cleared. This shows that households in Punjab and Sindh are more conscious about the adverse health impact of solid waste as compared to the households in Khyber Pakhtunkhwa and Baluchistan. Therefore, the households in Punjab and Sindh pay more cash for waste disposal services as compared to other provinces.

If households own the house, they pay more cash for waste collection and disposal services. This is both true in case of Pakistan and urban areas (Table 2). However, in rural areas, ownership of house did not provide enough evidence to capture any meaningful expression with households' cash payment for waste disposal. However, if number of rooms in a rural household house increase, then they pay more cash for waste collection and disposal. This is because most of the households in rural areas live in combine family system with large families. Their households' waste is huge in quantity and they needed services on cash for its disposal. Therefore, their cash payment for waste disposal and collection increases. Similar results can be seen in case of Khyber Pakhtunkhwa as people of this province mostly live in combine families and large household sizes (Table 3). Province wise breakdown reveals that only in Punjab, there is positive and significant impact of ownership of house on cash payment (Table 3). It means that if households own a house they pay more cash for waste collection and disposal. Results are insignificant on all other provinces. Unlike Pakistan and urban areas, households pay less cash in Sindh and Baluchistan if they live in rented houses. It means households are less bother to pay cash for waste disposal and collection because they need pay rent with other expenses. Similarly, if number of rooms increase in a house, the households cash payment decreases in Sindh. In other studies, Adzawla et al. (2019) linked the housing characteristics with households' decision to adopt a particular solid waste disposal system and found a negative impact of own housing on households' decision to adopt a particular solid waste disposal system. Similarly, Ahmed (2022) linked house ownership with households willingness to pay for improved solid waste management services and found a positive impact of house ownership on the willingness to pay for improved solid waste management services.

Dwelling type of households negatively but significantly affect the households cash payment for waste disposal and collection. For instance, if households live independently (unlike in joint family), they pay less cash for waste collection and disposal (Table 2). Households who live independently they carry on all responsibilities including the waste management. They can easily dispose-off waste on approved places and can save cash. This is true in both Pakistan and urban areas. Similar results can be seen for dwelling type such as households who live in apartments, part of large unit and compound. Almost similar results can be seen regarding various type of dwelling in four provinces. For instance, dwellers of independent houses pay less cash for waste collection and disposal in Sindh and KPK. Usually management of apartments, large units (of residential areas) and compounds manage all the wastes. In such cases, residents of such dwelling type do not need to pay cash for waste disposal. The results also show that residents of apartments in Punjab and Khyber Pakhtunkhwa, residents of large units of Sindh and Khyber Pakhtunkhwa and households of compounds of Sindh and KPK pay significantly less cash for waste collection and disposal (Table 3). In other studies, Adzawla et al. (2019) linked the dwelling type with households' decision to adopt a

particular solid waste disposal system and found a positive impact of compound housing on households’ decision to adopt a particular solid waste disposal system.

Urban households in Pakistan, Punjab, and Khyber Pakhtunkhwa pays significantly less cash payment for waste disposal as compared to counterfactual (e.g. rural households). In urban areas of the country, especially in the urban areas of Punjab and Khyber Pakhtunkhwa a well-developed system of waste collection and disposal is available to the households. This system properly works under the supervision of waste management companies and water and sensation agencies. Therefore, the urban households pay less cash for waste disposal as compared to rural households. However, in Sindh the opposite results are true. In Sindh, urban residents pay significantly more cash payment for waste disposal as compared to rural households. The system of waste collection and disposal available to households in Sindh is not organized nor efficient as compared to Punjab and Khyber Pakhtunkhwa. Therefore, the urban households in Sindh pay more cash for waste disposal as compared to rural households. In other studies, Adzawla et al. (2019) linked the regional dummies with households’ decision to adopt a particular solid waste disposal system and found ambiguous impact of regional dummies on households’ decision to adopt a particular solid waste disposal system.

The study also compared the cash payment of different provinces to better understand the picture. For instance, households in Punjab and Sindh significantly pay less cash for waste disposal services as compared to reference category, Khyber Pakhtunkhwa (Table 2). Similarly, urban and rural households in Punjab and Sindh pay less cash for waste disposal services as compared to households in Khyber Pakhtunkhwa. In case of Baluchistan, urban households cash payment for waste disposal services significantly lower as compared to urban dwellers of Khyber Pakhtunkhwa while results are insignificant for rural areas. As compared to other provinces the Khyber Pakhtunkhwa is rich in natural beauty and thus have abundant natural recreation sites and clean cities such that Peshawar is called the city of flowers and district Swat is called the Switzerland of Asia. For keeping the clean and neat environment of the beautiful natural sites and cities the households in this province pay more cash for waste collection and disposal services as compared to the households in other provinces.

4 Conclusion and recommendations

This study aims to document the existing solid waste collection and management system available to households in Pakistan. The study also explores the factors affecting the households cash payments for waste collection and disposal services. For accomplishing these objectives, the study used data of 16,155 households from Pakistan Social Living Measurement Survey (PSLM) for the year 2019-2020. Results of robust least square regression show that income has a significant positive effect on the households’ cash payment for solid waste collection and disposal services in Punjab whereas it has a significant negative effect on the households’ cash payments in Sindh. Punjab is the largest province of Pakistan by population, the province has relatively developed agriculture, industrial and services sector as compared to other provinces. Therefore, the households in this province give proper attention to waste management and hence their cash payment for waste collection and disposal increases as their income increases. Unlike Punjab, the households in Sindh is relatively poor as compared to the households in Punjab. Therefore, any increase in the households’ income decrease their payment on waste disposal services while increase their expenses on other essentials like food items, fuel, utilities, footwear, clothing, housing, education, and health. The efforts to increase the

households' incomes at national and provincial level such that providing subsidies or reducing taxes on food items, fuel, utilities, footwear, clothing, housing, education, and health can divert their attention to waste disposal issues and can increase their payments for waste collection and disposal services.

Moreover, if van/cart collect the waste from door, households' cash payment for waste collection and disposal increase both positively and significantly in Pakistan, urban areas, Punjab, Sindh and Baluchistan, respectively. This laid responsibility on municipal corporations, waste collection agencies, water and sanitation corporations and other private waste collection companies to further strengthen the van/cart facilities that the households dispose their waste at door and hence in return they will get batter cash for this service. However, we found opposite story in rural areas and in Khyber Pakhtunkhwa (KP) where the same facility at door reduce the households cash payments. Since large proportion of waste of rural households and KP households consisted of animal dung and households prefer to throw their waste in their agricultural field rather than to give to waste disposal cart/van. Therefore, alternative waste management policies are needed for these households which enable them to convert their waste to organic fertilizers.

Besides, if the bin or dumpster is present in the nearby locality, households' cash payment significantly reduces in Pakistan, urban areas, Sindh and KP. This show that the public bin availability is not enough to solve the waste management problems in the country and to increase the cash payment for waste collection. The real problem of waste management in the country is the distance from the waste disposal facility and the duration of clearness of waste disposal facility. For an instant, it is evident from the results that in Pakistan and urban areas if the waste disposal facility sites are situated near to the residential sites, the households cash payment for waste collection and disposal services increase, if this distance increases the households cash payment gradually falls. Therefore, it is recommended that the authorities put the bin near to the households' residence sites that they are dispose their waste in lesser time and lesser efforts. The second problem of waste management, is these bins are emptied/cleared by the concern person(s) or local authority(s) after few days. If the bin is not emptied on time than it can cause various diseases and can adversely affect the households' health. Therefore, it is recommended that the authorities must devise proper and quick method for clearing the bin every day.

Furthermore, if households own the house, they pay more cash for waste collection and disposal services. This is both true in case of Pakistan, urban areas and Punjab. Unlike Pakistan, urban areas, and Punjab, households pay less cash in Sindh and Baluchistan if they live in rented houses. It means households are less bother to pay cash for waste disposal and collection because they need pay rent with other expenses. The house ownership can significantly increase the households cash payment for waste collection and disposal services. The "Naya Pakistan Housing Scheme" is a best initiative of government of Pakistan in providing own housing facilities to poor and middle income households. This laid responsibility on the "Naya Pakistan Housing and Development Authority (NAPHDA)" to further strengthen and ease the process of loans for constructing and purchasing of houses. It will not only reduce the rent burden on households but also increase their cash payments for waste disposal and collection services.

5 Conflict of Interest

The authors declare that the research was conducted in the absence of any commercial or financial relationships that could be construed as a potential conflict of interest.

6 Author Contributions

"Conceptualization, G.M, G.R.M and B.A.; methodology, G.M. A.T.; software, G.M. N.H. and; validation, G.M., B.A. and N.H; formal analysis, G.M., A.T. and N.H.; investigation, G.M.; resources, B.A.; data curation, N.H,; writing—original draft preparation, G.M, B.A; writing—review and editing, B.A. A.T.,; visualization, G.M.; supervision, G.M. and A.T.; project administration, , N.H; funding acquisition, B.A. All authors have read and agreed to the published version of the manuscript."

7 Funding

This research was funded by Researchers Supporting Project Number (RSP2024R443), King Saud University, Riyadh, Saudi Arabia.

8 Acknowledgments

Authors are acknowledge King Saud University and University of Education, Lahore for supporting this research.

9 Reference styles

Abarca-Guerrero, L., Maas, G. and Hogland, W. (2015). Solid waste management challenges for cities in developing countries. Revista Tecnología en Marcha, 28(2), 141-168.

ADB. (2022). Solid waste management sector in pakistan: a reform road map for policy makers. (TCS220086-2). 6 ADB Avenue, Mandaluyong City, 1550 Metro Manila, Philippines: Asian Development Bank.

Adeleke, O., Akinlabi, S.A., Jen, T.C. and Dunmade, I. (2022). Environmental impact assessment of the current, emerging, and alternative waste management systems using life cycle assessment tools: a case study of Johannesburg, South Africa. Environmental Science and Pollution Research, 29(5), 7366-7381.

Adzawla, W., Tahidu, A., Mustapha, S. and Azumah, S.B. (2019). Do socioeconomic factors influence households' solid waste disposal systems? Evidence from Ghana. Waste Management & Research, 37(1), 51-57.

Afroz, R., Tudin, R., Hanaki, K. and Masud, M.M. (2011). Selected socio-economic factors affecting the willingness to minimise solid waste in Dhaka city, Bangladesh. Journal of Environmental Planning and Management, 54(6), 711-731.

Ahmed, S. (2022). Evaluation of Factors Determining the Household's Willingness to Pay for Improved Solid Waste Management Services: The Case of Logia Town. British Journal of Environmental Sciences, 10(4), 47-58.

Akhtar, S., Ahmad, A.S., Qureshi, M.I. and Shahraz, S. (2017). Households willingness to pay for improved solid waste management. Global Journal of Environmental Science and Management, 3(2), 143-152.

Bennett, L. (2008). Assets under attack: metal theft, the built environment and the dark side of the global recycling market. Environmental law and management, 20, 176-183.

Chukwuone, N.A., Amaechina, E.C. and Ifelunini, I.A. (2022). Determinants of household's waste disposal practices and willingness to participate in reducing the flow of plastics into the ocean: Evidence from coastal city of Lagos Nigeria. Plos one, 17(4), e0267739.

Dastjerdi, B., Strezov, V., Kumar, R., He, J. and Behnia, M. (2021). Comparative life cycle assessment of system solution scenarios for residual municipal solid waste management in NSW, Australia. Science of The Total Environment, 767, 144355.

Ekperi, P., Ude, O. and Ojeniyi, S. (2019). Analysis of Socio-Economic Factors Affecting Willingness to Pay for Solid Waste Disposal Services. J Waste Manag Disposal, 2(3), 1-7.

Endalew, B. and Tassie, K. (2018). Urban households' demand for improved solid waste management service in Bahir Dar city: A contingent valuation study. Cogent Environmental Science, 4(1), 1426160.

Hoornweg, D. and Bhada-Tata, P. (2012). What a waste: a global review of solid waste management. No.15. Washington, DC: World Bank.

Kaza, S., Yao, L., Bhada-Tata, P. and Van Woerden, F. (2018), What a waste 2.0: a global snapshot of solid waste management to 2050. World Bank Publications: Washington, DC, USA.

Khair, H., Rachman, I. and Matsumoto, T. (2019). Analyzing household waste generation and its composition to expand the solid waste bank program in Indonesia: a case study of Medan City. Journal of Material Cycles and Waste Management, 21(4), 1027-1037.

Khandelwal, H., Dhar, H., Thalla, A.K. and Kumar, S. (2019). Application of life cycle assessment in municipal solid waste management: A worldwide critical review. Journal of cleaner production, 209, 630-654.

Mulat, S., Worku, W. and Minyihun, A. (2019). Willingness to pay for improved solid waste management and associated factors among households in Injibara town, Northwest Ethiopia. BMC research notes, 12(1), 1-6.

Pan, Y., Li, M., Guo, H., Li, Y. and Han, J. (2022). Influencing factors and reduction of domestic solid waste at university dormitory in Shanghai, China. Scientific reports, 12(1), 1-12.

Pattnaik, S. and Reddy, M.V. (2010). Assessment of municipal solid waste management in Puducherry (Pondicherry), India. Resources, Conservation and Recycling, 54(8), 512-520.

Rahji, M.A.Y.and Oloruntoba, E.O. (2009). Determinants of households' willingness-to-pay for private solid waste management services in Ibadan, Nigeria. Waste management & research, 27(10), 961-965.

Omotayo, A.O., Omotoso, A.B., Daud, A.S., Ogunniyi, A.I. and Olagunju, K.O. (2020). What drives households' payment for waste disposal and recycling behaviours? Empirical evidence from South Africa's General Household Survey. International Journal of Environmental Research and Public Health, 17(19), 71-88.

Oyekale, A.S. (2015). Factors explaining households' cash payment for solid waste disposal and recycling behaviors in South Africa. Sustainability, 7(12), 15882-15899.

Tassie, K. and Endalew, B. (2020). Willingness to pay for improved solid waste management services and associated factors among urban households: One and one half bounded contingent valuation study in Bahir Dar city, Ethiopia. Cogent Environmental Science, 6(1), 1807275.

Tayyab, A., Ahmad, Z., Mahmood, T., Khalid, A., Qadeer, S., Mahmood, S., Andleeb, S. and Anjum, M. (2019). Anaerobic co-digestion of catering food waste utilizing Parthenium hysterophorus as co-substrate for biogas production. Biomass and Bioenergy, 124, 74-82.

Vassanadumrongdee, S. and Kittipongvises, S. (2018), Factors influencing source separation intention and willingness to pay for improving waste management in Bangkok, Thailand. Sustainable

Environment Research, 28(2), 90-99.
