# Supplementary material for: Households’ expenditures for solid waste management services: Influencing factors and deep insight
Source: PLoS One. 2025 Jun 23;20(6):e0324085. doi: 10.1371/journal.pone.0324085 (PMC12184931; doi:10.1371/journal.pone.0324085)
Supplement: Attachment — Submitted filename: Response to Reviewers.docx [file pone.0324085.s004.docx]

**Editorial Team Comment:** In addition to the criticisms below, this typo also needs to be corrected.  (Bennetst, 2008) at the line 72.

**Response to Editorial team:** We address this typo.

**Editorial Team Comment:** Please include the following items when submitting your revised manuscript:

A rebuttal letter that responds to each point raised by the academic editor and reviewer(s). You should upload this letter as a separate file labeled 'Response to Reviewers'.

**Response to Editorial team:** There is rebuttal from authors. All comments are addressed.

**Editorial Team Comment:** A marked-up copy of your manuscript that highlights changes made to the original version. You should upload this as a separate file labeled 'Revised Manuscript with Track Changes'.

**Response to Editorial team:** Marked-up copy is provided that highlighted changes.

**Editorial Team Comment:** An unmarked version of your revised paper without tracked changes. You should upload this as a separate file labeled 'Manuscript'.

**Response to Editorial team:** It is provided

**Editorial Team Comment:** When submitting your revision, we need you to address these additional requirements.

1. Please ensure that your manuscript meets PLOS ONE's style requirements, including those for file naming.

**Response to Editorial team:** It is provided

**Reviewers' comments:**

**Reviewer's Responses to Questions**

**Comments to the Author**

1. Is the manuscript technically sound, and do the data support the conclusions?

The manuscript must describe a technically sound piece of scientific research with data that supports the conclusions. Experiments must have been conducted rigorously, with appropriate controls, replication, and sample sizes. The conclusions must be drawn appropriately based on the data presented.

Reviewer #1: Yes

Reviewer #2: Partly

**Response to Reviewers:** We re-regress the model, clarified the data and wrote findings and hence conclusion based on these.

2. Has the statistical analysis been performed appropriately and rigorously?

Reviewer #1: Yes

Reviewer #2: No

**Response to Reviewers:** We re-regress the model and provided its justifications.

3. Have the authors made all data underlying the findings in their manuscript fully available?

The PLOS Data policy requires authors to make all data underlying the findings described in their manuscript fully available without restriction, with rare exception (please refer to the Data Availability Statement in the manuscript PDF file). The data should be provided as part of the manuscript or its supporting information, or deposited to a public repository. For example, in addition to summary statistics, the data points behind means, medians and variance measures should be available. If there are restrictions on publicly sharing data—e.g. participant privacy or use of data from a third party—those must be specified.

Reviewer #1: Yes

Reviewer #2: Yes

**Response to Reviewers:** Thank you for appreciating.

4. Is the manuscript presented in an intelligible fashion and written in standard English?

PLOS ONE does not copyedit accepted manuscripts, so the language in submitted articles must be clear, correct, and unambiguous. Any typographical or grammatical errors should be corrected at revision, so please note any specific errors here.

Reviewer #1: Yes

Reviewer #2: No

**Response to Reviewers:** We re-write the paper. Paper quality is improved based on your comments. Please let us know how we can improve it further.

**5. Review Comments to the Author**

**Reviewer #1:**

**Reviewer #1:** The manuscript offers valuable insights into Pakistan's waste management system, and the statistical analysis is both thorough and satisfying. However, I have highlighted some areas in the attached document where corrections are necessary. Firstly, the general statistics provided in the introduction need clearer and more precise references. Additionally, some figures should be redesigned to enhance clarity and visual impact.

**Response to Reviewer:** Thank you very much for reviewing our paper. We addressed all comments and there is no rebuttal. We cited all the statistics and revised the Figures under your valuable comments.

**Reviewer Comment:** This beginning is a kind of standard beginning for an introduction. Do we really need to refer to another paper?

**Response to Review:** We revised the introduction based on your comment. In opening of this paragraph two (Line 46-60). We changed it based on your and other reviewers’ comments. The study highlights some factors that cause solid waste. Therefore, we think these claims should be properly cited. Moreover, we added new opening of introduction part. Please see Lines 33-44.

**Reviewer Comment:** The rest of the paragraph provides statistics on the topic. However, based on the referencing, it may be misunderstood that the authors are the source of these statistics. For instance, it is mentioned that the annual global generation of solid waste exceeds 2 billion tonnes; however, this statistic actually comes from a World Bank study. Therefore, it would be more accurate to emphasize that the data is sourced from the World Bank within the text.

**Response to Reviewer:** We incorporated this change. See line 46-57

**Reviewer Comment:** "I recommend adding a paragraph that outlines the structure of the study. For example:

The remainder of the paper is organized as follows: ...."

**Response to Reviewer:** We incorporated this change. See last paragraph of introduction from line 148 to 151.

**Reviewer Comment:** This figure is somewhat confusing. I suggest dividing it into two separate figures. The first (figure 1.a.) could display Pakistan – Urban – Rural (perhaps using a pie chart). The second figure (figure 1.b.) could focus on the provinces, which could be represented using either a bar chart or another pie chart.

**Response to Reviewer:** We incorporated this change. Please see Figs 2, 3, 4 and 5.

**Reviewer comment:** "Once again, I believe the figure should be divided. If it’s not divided, then the colors used in the figures must be designed consistently for clarity."

**Response to Reviewer:** We incorporated this change. Please see Figs 2, 3, 4 and 5.

**Reviewer comment:** Same comment like previous two comment.

**Response to Reviewer:** We incorporated this change. Please see Figs 2, 3, 4 and 5.

**Reviewer #2: Reviewer Comments**

Summary

This paper addresses an important environmental issue of our time- solid waste management in a developing country context. It sought to contribute to knowledge on the factors that determine household payment for solid waste management. The authors cited related studies from different countries. The authors used secondary national data for the study. The findings from this study will contribute to policy and dialogue on solid waste management in Pakistan and similar developing countries. However the paper needs to clearly establish the gap in knowledge it seeks to fill and state the objectives more clearly. It also needs to be better organized in terms of methodology, presentation and grammar.

**Response to Reviewer:** Thank you very much for reviewing our paper. We really learnt from your comments. We are happy to incorporate those changes and there is no point of rebuttal. We tried to establish gap in the literature (See highlighted parts of introduction) and we also clarified our objectives of the study (See line 143-146). We improved our methodology based on your comments (See methodology part) and presented findings of our study based upon the information gathered as a result of the methodology (See Results and Discussion).

**Abstract**

**Reviewer Comment:** The abstract is a good summary of paper.

**Authors Response:** Thank you for appreciating the Abstract

**Reviewer Comment:**

Introduction

The authors need to describe a little bit more of the existing waste management system in Pakistan. Is it public sector led or private sector led? Is there synergy between public sector and public sector in waste management? What policy directions does the government currently pursue for waste management. That will give the reader a better context for the paper. The objective should be stated more explicitly. To ‘document’ waste system as an objective, should be restated.

**Response to Reviewer:** We address this comment and revised our introduction based on your comment. Solid waste collection is owned and operated by government of Pakistan. However, in some metropolitan cities public-private partnership is there. We provide the complete synergy between public and private sector. Revised introduction can be seen in first paragraph of introduction (Lines 33-44). Waste management system in Pakistan and its four province along with public-private partnership can be seen in Paragraph 6, 7, 8 and 9 of Introduction (Lines 94-131). The objectives are restated and clarified (Please See line 143-146).

**Reviewer Comment:**

Manuscript Formatting

The next section after introduction is labelled “Manuscript Formatting’ and this appears like a misnomer. The authors should crosscheck if that is what the authors really want to name that section.? It seems materials and methods or methodology should be a better option.

**Response to Reviewer:** Thank you very much for highlighting typo. We changed it into Materials and methods

**Reviewer Comment:** The study Area should be a sub- section in this Materials and Methods or Methodology section. Here the authors should describe the area of study briefly. How many provinces in all are in Pakistan? Was the data used across the whole of Pakistan? If the data is a nationally representative data, then the authors should discuss Pakistan socio-demographics relevant to their study. What are the sources of livelihood in the different provinces, especially the rural areas that earn more income than urban areas. Also a map of the country where the major provinces highlighted in the results section is distinctively shown would be appropriate.

**Response to Reviewer**: We address these comments by following ways;

a. The study Area is provided under Materials and Methods section. We also described the area of study. See Lines 154to 192.
b. We briefly described Pakistan and its four provinces. The data used in the study represent the whole Pakistan. The data is nationally represented that is collected through House-hold Integrated Economic Survey (HIES) 2018-2019. We briefly described the socio-economic status of Pakistan and its four provinces under the heading “Study Site”. We also provided livelihood status of different provinces (See Paragraph 2 and 3 under heading “Study Site”). See Lines 154 to 192
c. Map of the study showing different province is also provided. Please see Fig 1.

**Reviewer Comment:**

Data

This section needs to describe the data set a bit more. How were the samples chosen by the Bureau of statistics?. Is this the only wave available for Pakistan ? Why did the authors not use panel data if there are other waves?.

**Response to Reviewer:** We address this comment in the first paragraph under the heading “Data” and briefly justify here. This study used the recent data of the Pakistan Social Living Measurement Survey (PSLM) for the year 2019-2020, conducted by the Pakistan Bureau of Statistics. PSLM, 2019-20 is the twelfth round of a series of surveys, initiated in 2004. The first round of the survey started in 2004-2005 and up to date the recent most round survey data is available for the year 2019-20. However, the survey is not carried out for two round such that for 2009-10 and for 2017-18. Besides in each round the sample size is changed. Thus, making a panel data from various round of PSLM is not possible. Thus, we use the most recent data of the 12th round of PSLM for the year 2019-2020, which include 160,654 households throughout Pakistan. However, some households did not report the information on their cash payments for waste collection and disposal services, so after organizing the data, we used data from 16,155 households who pay cash for waste collection and disposal services. For further detail, please see Lines 197-213.

**Reviewer Comment:**

Empirical Methods

This section presented by the authors seems to be the analytical framework. What about the empirical strategy? The authors should specify the model – robust least squares regression they used. What is your identification strategy for establishing causality? The authors should cross check what gi is in the various equations where it is specified and make sure there are no errors. It assumes different variables in the two equations where it is specified.

**Response to Reviewer:** We added sub-heading under Material and Methods namely; Analytical Framework and Estimation strategy in the light of your comment. We incorporate these under heading of Analytical Framework. Please see Lines 224-227 and 236-237.

**Reviewer Comment:** The authors should reconsider this empirical method section in terms of model used……. There are a few socio-demographic variables expected to be included like education of household head, gender of household head, number of income earners in household which the authors did not include and did not provide reasons for non-inclusion.

**Response to Reviewer:** We address this comment under the Sub-subheading of empirical estimation strategy. Please paragraph 2, 3 and 4 (See line 250-253, 263-296) under Empirical Estimation Strategy. We also address your comment regarding education, gender, number of income earners in the last paragraph of Conclusion.

**Results and Findings**

**Reviewer Comment:** The first figure is not part of your findings. It should be moved to the data section, under sampling. Figure labels are usually placed after the figure not before the figure. This style runs through all the figures the authors presented and should be corrected. Figure 2 label should go below the figure. It appears it is mean cash payment. If so then it should be reflected in the figure label.

**Response to Reviewer:** We moved this Figure in Material and Methods section Please see Figure 1 and 2. Similar comment given by other reviewer so we break this Figures into two figures. Moreover, we used journal style for figures and tables.

**Reviewer Comment:** The mean household payment for waste collection should come after Mean household income. Did the authors try to explore why monthly mean income is higher in rural areas than urban areas from literature. It would be good to state it here, because the reverse is expected. Also the authors should explore why payment for waste is higher in rural areas from literature. Apriori we expect rural areas to be agriculture based, and to use decomposable waste for home gardens, therefore minimizing waste quantity to be discarded, and associated cost. The authors should try to find from more microlevel waste study in Pakistan why there is higher payment in rural areas aside from the reasons they gave.

**Response to Reviewer:** Mean cash payment is added. Please see Figure 6. Moreover, we address other part of comment in first paragraph of Results and Discussion under the subheading “Existing solid waste collection…”. See line 306-314.

**Reviewer Comment:** For the descriptive statistics, it would be good to include the minimum and maximum values of the variables.

**Response to Reviewer:** We included minimum and maximum values of the variables. Please see Table 1.

**Reviewer Comment:** Was the household waste management techniques mutually exclusive? Like a household using public waste collector did not at the same time use private? A household using the above two did not have a bin accessible to them?

**Response to Reviewer:** We incorporated this comment from Line 366 to Line 376.

**3.3 Factors affecting cash payments**

The authors need to be careful in drawing inferences from the data. For example the authors state that ‘ However, in Punjab (Table 3) we observed that an increase in household income increase their cash payment for solid waste collection and disposal services whereas in Sindh an increase in household income brings a significant decrease in their cash payments for waste collection and disposal services. Punjab is the largest province of Pakistan by population, the province has relatively developed agriculture, industrial and services sector as compared to other provinces. Therefore, the households in this province give proper attention to waste management and hence their cash payment for waste collection and disposal increases as their income increases. Conversely, by population Sindh is the second largest province of the country, the province is poor as compared to the Punjab Therefore, any increase in the households’ income decrease their payment on waste disposal’ see lines 286-294. Instead state that ‘maybe due to……., or could be because…….’ Cross check every where in this section for similar inferences.

**Response to Reviewer**: We address this comment. Please see Line 360-369.

**Reviewer Comment:** Line 341 contradicts what you stated in earlier section that rural households are richer see line 186-187.

**Response to Reviewer:** Contradictions are removed. Please see Lines 305-313 and 513-518.

The authors need to take another look at the interpretation of their data. It goes back to the empirical strategy given the nature of their data. There are many categorical explanatory variables in the data. They should explore a more suitable model for analysis.

**Response to Reviewer:** We address this comment under the heading of analytical framework (Lines 223-248) and estimation strategy (See lines 251-296). According to these model we interpreted our results. For instance, previous literature (see references study 1 and 18) used binary dependent variable (yes = 1 and 0 otherwise), however, in our model the dependent variable is continuous in nature (household monthly cash payment for waste collection and disposal services in Pakistani rupees). The nature of the dependent variable naturally suggests estimation of liner regression to determine the factors affecting waste disposal through cash payments.

**Conclusion and recommendation**

**Reviewer Comment:** The authors essentially repeated almost verbatim what they stated in the results section. The conclusion should be a brief summary of the study highlighting study objectives, empirical approach and major findings then the recommendations which should inform policy direction.

**Response to Reviewer:** We revised the whole Conclusion and Recommendation part. We briefly provided summary of the study highlighted objectives and findings. Based the empirical approach and major finding we provided recommendation. We removed unnecessary points based on imagination and focused on main findings. Please see whole Conclusion part.

References

**Reviewer Comment:** Some cited papers are not in reference list. Example – Kaza and Lisa 2018; Tadesse et al 2007. The authors should consider providing the DOI of the papers.

**Response to Reviewer:** We used journal reference style and all cited material is refered in the reference list and vice versa. All DOIs are provided where available.

**Major issues**

**Reviewer Comment:** The authors should state the empirical model used, review if that empirical model best suits the data available. They should also cross check the interpretation of the results.

**Response to Reviewer:** We re-checked our model, gave its justification based on past studies and proved that selected model is good-fit. Based on selected model, we regress the results and interpret accordingly. Please let us know which model we should further use.

Minor Issues

**Reviewer Comment:** The authors should consider enlisting the services of a writing coach to improve the grammar and flow of the write-up.

**Response to Reviewer:** We re-write the paper. Paper quality is improved based on your comments. Please let us know how we can improve it further.
