# Supplementary material for: Households’ expenditures for solid waste management services: Influencing factors and deep insight
Source: PLoS One. 2025 Jun 23;20(6):e0324085. doi: 10.1371/journal.pone.0324085 (PMC12184931; doi:10.1371/journal.pone.0324085)
Supplement: Attachment — Submitted filename: Response_to_Reviewers_auresp_2.docx [file pone.0324085.s005.docx]

**Reviewer #1: Dear Authors,**

I have reviewed the revised manuscript and observed that all the requested revisions have been addressed thoroughly. Thank you for your effort and attention to detail. I recommend the manuscript for publication.

Response: Dear reviewer thank you for your appreciation.

**Reviewer #2: General Comments:**

1. The authors have revised the work based on earlier comments, but a few comments were not effected, like adding some more variables in the regression that was suggested. They added it in conclusion as suggestion for further study.

**Response:** Special thanks for this comment, as per your suggestion, we included socio-demographic variables like education of the household head, age of the household head, gender of household head, and number of income earners in the household. The inclusion of these variables furthers improve the regression results (for details see lines 259-262; 420-424; 448-466; 472-489; 493-543; 546-555; 564-569; 572-597; 603-605; 609-611; 614-617; 621-624; 639-642; 653-658 Table 1; Table 2; and Table 3). We also revised the conclusion and recommendation part based on new results.

2. In revising the work, the authors paid little attention to the grammar and sentence structure. A few examples of corrections that need to be done follow:
   Line 36, remove Now a days. Start the sentence with ‘Developed and developing countries……

**Response:** Incorporated. Please see Line# 37

Line 46: ….. the demand for solid waste management services has increased. ( insert ‘services’ after waste management)

**Response:** Incorporated. Please see Line# 47

Line 49:…… billion in 2025; with this, the waste generation….. ( insert ‘with’ before this the waste management…).

**Response:** Incorporated. Please see Line# 50

Line 56: in developing countries…….. 50 percent of municipal operation budget is spent on… 9
(insert ‘is’ before spent on…)

**Response:** Incorporated. Please see Line# 57

Line 78- 79

**Response:** Please let us know what is issue in these lines?

Line 83-85: users of solid waste management services pay higher amount than ???? for waste management ( something is missing there. If you cant supply that information, then remove that last sentence.

**Response:** We removed this sentence.

Line 86: usually people are reluctant to pay for waste disposal….(insert ‘are’)

**Response:** Incorporated. Please see Line# 84

Line 136: Infrastructure which create serious environmental….. (replace creating with create)

**Response:** Incorporated. Please see Line# 135

Line 138: The country is facing enormous challenges …( insert is after country)

**Response:** Incorporated. Please see Line# 137

Line 145: Findings of this study will guide the policy makers in …( remve provide help)

**Response:** Incorporated. Please see Line# 144

Line 148:

**Response:** Please let us know what is issue in this line?

Line 152. Please choose Methodology instead of materials and Methods. ( materials and methdos are usually for laboratory based research work.

**Response:** Thank you very much for this comment. It is the learning of a day for me. Incorporated. Please see Line# 151
Line 156: in the country, 61 percent …population live in rural areas ( use live instead of living, make same changes in line 157)

**Response:** Incorporated. Please see Line# 155 and 156

Line 159 Remove the statements after the Arabian Sea and Golf of Oman in the south. End the sentence here and delete the rest of information on countries bothering Pakistan.
Further summarize the study site please.

**Response:** We incorporated these changes and summarized this part. Please see lines#153-159.

Line 199-201: recast as follows: The first round of the survey started in 2004-2005 and upto date the most recent round of survey data was conducted in year 2019-20. The survey was not carried out for 2 rounds -2009-10 and for 2017-18. ( Please change your sentence with this suggested).

**Response:** Incorporated. Please see Lines # 200-202. Thank you for very nice suggestion.

Line 300: form fig 4 we… ( change Form to from)

**Response:** Incorporated. Please see Line# 302

Line 306: …first, Rural incomes ( remove capital ;R” in Rural and use small ‘r’. cross check other sentences for such cases in the entire work.

**Response:** Incorporated. Please see Line# 308. Whole manuscript is cross check and incorporated the changes where necessary.

Line 338: the results can have two compensations ( remove compensation and replace with ‘possible interpretations’)

**Response:** Incorporated. Please see Line# 340

Line 341; …. This might be the reason that there is well established ( I suggest ….’It could be because there is well established…).

**Response:** Incorporated. Please see Line# 343-344

Line 345: Secondly, waste management is a need rather than choice… ( I suggest: waste management service is important, so people spend money for it…)

**Response:** Incorporated. Please see Line# 347-348

Line 347-348 ( is Akhter’s finding in relation to a month or a year? It is good to state whether it is USD 4.8 per month or per year.)

**Response:** Akhtar’s study is based survey data in elite areas of Lahore. Although he did not mentioned either it was monthly or yearly but we perceived from his study it is monthly expenses on waste disposal services. See Line# 349-350

Line 370 -373 Please cross check the information in these sentences with what you have in the figure. In the discussion you have 83% with waste collection bin and 17% without waste collection bill, but your figure is saying the opposite. There is inconsistency. In figure 9, you have bin/ waste collection point not available/accessible 83% but the arrow is pointing to the small portion of the pie. You have bin/waste collection point is available /accessible by 13% but the arrow is pointing to larger portion of the pie.

**Response:** We revised the figure 9. Thank you for highlighting it. See line# 372-375 as well.

Line 394 -397: is it possible for you to find the mean/ average time for all respondents to get to the bin/collection point? That will help you if really most spend a lot of time disposing waste. From literature, is there a recommended time to be spent disposing waste? Beyond which time it is regarded as too much time? If there is such information and you can cite it, it will make your discussion more interesting.

**Response:** Dear Reviewer, thank you for your insightful comment. Unfortunately, we are unable to calculate the mean or average time for all respondents to reach the bin/collection point because the data was collected using a Likert scale, which does not provide precise numerical values for such calculations. However, we acknowledge the importance of this aspect and we discussed relevant literature to offer insights into the time spent disposing of waste. (For details see line 386-391)

Line 439-617: please carefully make sentence corrections to convey intended meanings to readers.

**Response:** We revised major portion of manuscript based on new variables. We highlighted this in green. Please let us know where we can further improve it.

Line 437: Factors affecting the households cash payments for waste collection and
disposal services

**Response:** Our heading is exactly like this. Please let us know what correction required in “Factors affecting the households cash payments for waste collection and
disposal services”

3. The regression reported here is only the robust least squares. Is it possible to report the OLS that was tried before testing for heteroskedasticity. It can be reported in same tables, side by side with the robust least squares. It will help readers to appreciate what you stated about the OLS and the robust least squares.

**Response:** Special thanks for this comment, as per your suggestion, we reported the results of OLS side by side with robust least square (RLS) results in the two tables (for details see Table 2 and Table 3).

4. Please check out your sentences especially in the discussion of the regression results.

**Response:** We revised major portion of manuscript based on new variables. We highlighted this in green. Please let us know where we can further improve it.
